# Towards the Development of Global Nano-Quantitative Structure–Property Relationship Models: Zeta Potentials of Metal Oxide Nanoparticles

**DOI:** 10.3390/nano8040243

**Published:** 2018-04-15

**Authors:** Andrey A. Toropov, Natalia Sizochenko, Alla P. Toropova, Jerzy Leszczynski

**Affiliations:** 1 Laboratory of Environmental Chemistry and Toxicology, Department of Environmental Health Science, IRCCS-Istituto di Ricerche Farmacologiche Mario Negri, 20156 Milano, Italy; andrey.toropov@marionegri.it (A.A.T.); alla.toropova@marionegri.it (A.P.T.); 2 Interdisciplinary Center for Nanotoxicity, Jackson State University, Jackson, MS 39217, USA; sizochenko@dartmouth.edu; 3 Department of Computer Science, Dartmouth College, Hanover, NH 03755, USA

**Keywords:** zeta potential, QSPR, nano-QSPR, modeling, metal oxide nanoparticles, quasi-SMILES, CORAL, descriptors

## Abstract

Zeta potential indirectly reflects a charge of the surface of nanoparticles in solutions and could be used to represent the stability of the colloidal solution. As processes of synthesis, testing and evaluation of new nanomaterials are expensive and time-consuming, so it would be helpful to estimate an approximate range of properties for untested nanomaterials using computational modeling. We collected the largest dataset of zeta potential measurements of bare metal oxide nanoparticles in water (87 data points). The dataset was used to develop quantitative structure–property relationship (QSPR) models. Essential features of nanoparticles were represented using a modified simplified molecular input line entry system (SMILES). SMILES strings reflected the size-dependent behavior of zeta potentials, as the considered quasi-SMILES modification included information about both chemical composition and the size of the nanoparticles. Three mathematical models were generated using the Monte Carlo method, and their statistical quality was evaluated (*R*^2^ for the training set varied from 0.71 to 0.87; for the validation set, from 0.67 to 0.82; root mean square errors for both training and validation sets ranged from 11.3 to 17.2 mV). The developed models were analyzed and linked to aggregation effects in aqueous solutions.

## 1. Introduction

Massive production of nanoparticle-based materials results in their release into the environment. It is widely known that certain physical characteristics of nanoparticles, such as size, shape, charge, chemical composition, and the pH of the solution, may directly influence their toxicity [1,2,3,4]. Nanoparticles become involved in processes of dissolution, agglomeration, and settlement when released into the aquatic environment [5]. Changes in the stability or surface charges of nanoparticles in solution are known to induce toxic effects [6]. 

Zeta potential (*ζ*) could indirectly represent both stability and the surface charge of nanomaterial [6]. Zeta potential measurements are among the characteristics recommended for experimental testing of nanomaterials [7]. In general, experimental testing is expensive, so it is vital that robust theoretical approaches that can be applied for the preliminary computational modeling of various properties of nanomaterials [8]. One of the most popular approaches is the quantitative structure–property relationship (QSPR) technique [8]. The QSPR is used to establish links between features of the nanomaterial’s structure and the target property. The QSPR has been widely applied over the last decade to predict nanoparticle properties [8,9,10,11,12,13,14,15,16]. To date, only three research articles have reported QSPR modeling of zeta potentials [17,18,19]. These nano-QSPR models focused only on chemical composition-dependent descriptors [17,18,19]. In fact, these models include a variety of core compositions but do not take into consideration size-dependent effects (namely, the dataset do not contain information about the same core composition nanoparticles of different sizes). Zeta-potentials are size-dependent, so the inability of previously developed models to address this feature significantly lowers the transferability of these models.

In order to develop a global model, the size-dependent behavior of zeta potentials should be taken into account [20]. This would facilitate the creation of revised and boosted datasets that contain chemically diverse nanoparticles of varied sizes. However, a fundamental problem related to the scarcity and inconsistency of experimental data on zeta potentials should be the first to overcome. This problem appears in the connection to differences between synthesis and measurement protocols [21]. As a consequence, variations in measuring protocols may result in a significant variance in data and may lead to inaccuracy in data assessment. Thus, an established list of criteria should be followed during the development of databases suitable for the computational modeling. Based on that, the first aim of this research article is to gather experimental reports on zeta potential measurements and analyze the reliability of collected data points.

Apart from the reliability of sources with experimental data, the problem of computational characterization of nanoparticles still exists [1]. Nanoparticles are characterized by a high structural complexity. In most cases, traditional QSPR methods are unable to distinguish structural features of nanoparticles [11]. Addressing this issue, specific descriptors for nanoparticles have been recently developed [8]. Despite promising results, the creation of a unified and simple system of nanoparticle representation is an open question. The second aim of this research article is to create an universal system of representation that can be used to describe nanomaterials of different sizes. 

In the present study, the authors collected (using multiple literature sources) and curated 87 data points of zeta potential measurements in aqueous solutions for differently sized nanomaterials made of silica and metal oxides. Features of nanoparticles were represented using a modified version of the simplified molecular input line entry system (quasi-SMILES) [22,23,24]. Nano-QSAR models were constructed and provided the basics for a mechanistic interpretation of results. The supremacy of presented descriptors over other nano-descriptors was evaluated.

## 2. Materials and Methods 

### 2.1. Data Collection and Curation

The current study focuses on zeta potential measurements of of silicon- and metal oxide nanoparticles in water. The main obstacle we faced was the data curation, as the data from multiple sources was inconsistent and contradictory. Initial criteria assessed for literature search were as follows:

1. Zeta potentials were measured for no less than three different oxide nanoparticles.
2. Measurements were conducted for non-coated nanoparticles in pure water.
3. Core composition, nominal size, and the size of the aggregate in the water were reported.
4. Contradictory data points (reports of the same core composition and the same size, but with significant differences in zeta potential values) were removed.

Endpoints that meet abovementioned criteria are reported in [1,12,18,25,26,27,28,29,30,31,32,33].

### 2.2. Quasi-Simplified Molecular Input Line Entry System (SMILES) Optimal Descriptors and Model Generation

**Scheme 1:**
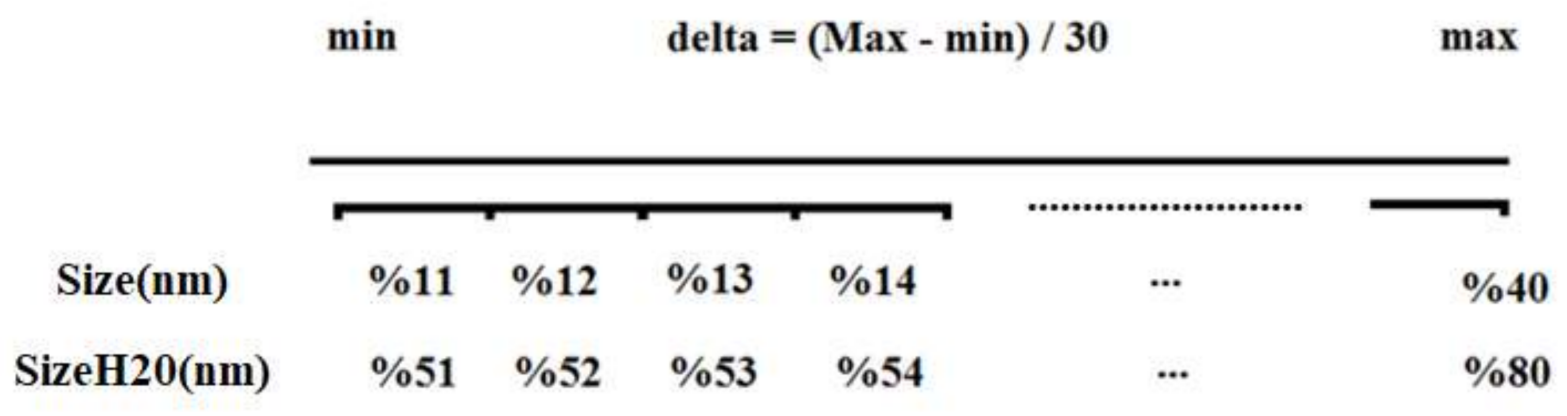
The scheme of converting variables from Table 1 into discrete parameters.

As mentioned in the introduction, features of the molecular architecture of metal oxide nanoparticles combined with the size-dependent behavior of the target property render traditional QSPR tools useless. At the same time, the model for such data could be built under the paradigm that an “endpoint is a mathematical function of all available eclectic data” [34,35,36]. This paradigm could be applied using a technique known as “quasi-SMILES.” The quasi-SMILES system of structural representation is a suitable tool to encode any available eclectic data. To represent nanoparticles’ structures using quasi-SMILES, the numerical parameters from Table 1 (the nominal sizes of nanoparticles and their sizes in H_2_O) were translated into discrete values, as presented in Scheme 1.

In the presented case, the optimal descriptor is a translator of eclectic information into the predictive model [37,38,39]. For instance, using Scheme 1, Al_2_O_3_ nanoparticles form the first row in Table 1 (a nominal size of 11.40 nm and a size in media of 94.70 nm) were attributed to the quasi-SMILE code O=[Al]O[Al]=O%11%51. For this data point, both nominal size and size in media lie in the first brackets of conversion (Scheme 1). Details about the assignment of attributes in quasi-SMILES are presented in the supplementary information (Table S1 and Figure S1 for the nominal size; Table S2 and Figure S2 for the size in water).

The collected dataset was split into training, invisible training, calibration, and validation sets. The training set was used to calculate correlation weights and to build the model. The invisible training set was aimed to determine whether the correlation between DCW(T*,N*) and the values of the zeta-potentials is satisfactory for structurally similar nanoparticles outside of the training set. DCW stands for the descriptor of correlation weights. The calibration set was evaluated to estimate the cutting value for the overtraining. The validation set was applied to estimate a predictive potential of a modeled.

The model was developed using the Monte Carlo approach [19,35,36]. Final models were represented by the following equation:

(1) $${\xi \;=\;C}_{0}{\;+\;C}_{1}*\mathrm{DCW}\left(T^{*}{,N}^{*}\right)$$

where *T* is the threshold, i.e., the integer to divide attributes of quasi-SMILES into two classes: (i) rare, if the number of an attribute is less than *T* in the training set, and (ii) frequent, if the number of an attribute is large or equal to *T*. *N* is the number of epochs of the Monte Carlo optimization. The DCW is calculated as

(2) $$\mathrm{DCW}\left(T^{*}{,N}^{*}\right)=\sum^{\text{​}}{\mathrm{CW}(A}_{k})$$

where *A*_k_ is an attribute of quasi-SMILES, and *T* and *N* are parameters of the Monte Carlo optimization.

The CW(*A*_k_) are correlation weights for different *A*_k_. The correlation weights were used to calculate the DCW(T*,N*) with a maximal value for the target function (TF):

(3) $${\mathrm{TF}\;=\;R}_{\mathrm{TRN}}{\;+\;R}_{\mathrm{iTRN}}\;-\;\left|R_{\mathrm{TRN}}{\;+\;R}_{\mathrm{iTRN}}\right|*Const$$

where R_TRN_ and R_iTRN_ are correlation coefficients between the optimal descriptor and zeta potential for the training and invisible training sets, respectively. *Const* is an empirical parameter that, in the current study, was set as 0.1.

The measure of statistical quality of attributes (*A*) from the model for a given split (i.e., training, invisible training, calibration, and validation sets) can be estimated via *defect(A)* as follows [36]:

(4) $$defect\left(A\right)=\{\begin{array}{c} \frac{\left|P_{\mathrm{TRN}}\left(A\right)-P_{\mathrm{iTRN}}\left(A\right)\right|}{N_{\mathrm{TRN}}\left(A\right)+N_{\mathrm{iTRN}}\left(A\right)},\;\mathrm{if}\;N_{\mathrm{iTRN}}\left(A\right)>0\\ 1,\;\mathrm{otherwise} \end{array},$$

Using data on *defect*(*A*) for all attributes of quasi-SMILES involved in building up the model, one can estimate defect of quasi-SMILES as

(5) $$defect\left(quasiSMILES\right)=\sum^{\text{​}}defect\left(A\right),\;A∊quasiSMILES.$$

If the given split is “good,” then *defect*(*A*) for all attributes of quasi-SMILES are equal to zero. In reality, for the majority of cases, *defect*(*A*) > 0. The average value of *defect*(*quasiSMILES*) (calculated for the training set) are used to separate quasi-SMILES into two categories: (i) the domain of applicability and (ii) outliers. 

### 2.3. Alternative Descriptors 

As mentioned in the introduction, the majority of currently available descriptors for both conventional organics and nanomaterials do not take into consideration size-dependent effects of nanoparticles [8]. We compared presented quasi-SMILES parameters with (a) quantum-chemical descriptors (calculated for small clusters as discussed in Mikolajczyk A. et al. [17]) and (b) ionic characteristics (calculated based on chemical formula as discussed by Sizochenko N. et al. [16]). 

## 3. Results and Discussion

We initially extracted more than 150 data points; however, after data curation (as is described in Materials and Methods section), we included in the reliable dataset 87 zeta potential measurements from 12 literature sources (Table 1) [1,12,18,25,26,27,28,29,30,31,32,33].

**Figure 1:**
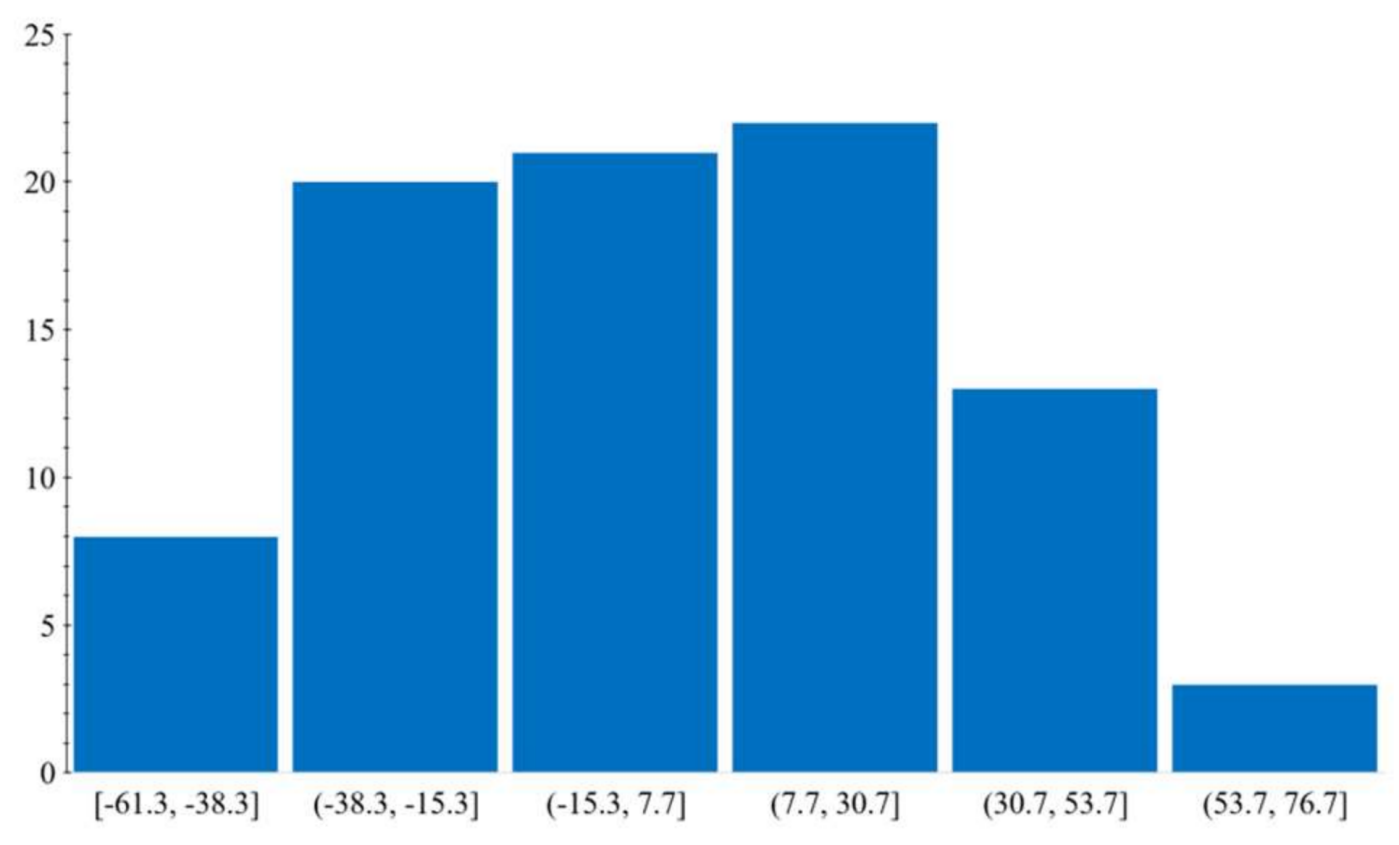
Distribution of zeta potential values.

The analysis of the distribution of zeta potential values in the collected dataset (Figure 1) shows that the data has an almost normal distribution with slightly skewed data points toward high positive values.

Predictive models were developed for three random splits of the data. Splitting of the initial dataset is presented in Table S3. Table 2 contains data on the correlation weights obtained by the Monte Carlo optimization procedure. As we can see, each model included different weights for the same attributes. Table 3 contains the statistical characteristics of developed quasi-SMILES based models.

As presented in Table 3, the statistical characteristics of the developed quasi-SMILES-based models were satisfactory. At the same time, all attempts to build models using quantum-chemical descriptors or ionic characteristics adopted from literature failed [17,18]. *R*^2^ for the training set for all non-quasi-SMILES models was below 0.47, which is a sign of random modeling. Indeed, quantum-chemical parameters are capable of representing size-dependent effects; however, in fact, the true power of quantum-chemical descriptors has never been identified, as authors have only conducted calculations for clusters of predefined size [9,17,18]. Similar situation is observed the ionic characteristics (*R*^2^ training < 0.5): it is clear that descriptors derived from the chemical formula alone are not capable of representing the size-dependent behavior of zeta potential.

Let us take a closer look at quasi-SMILES based models. According to the *defect*(*quasiSMILES*), the model for Split 1 contained 9 outliers in the training set and 1 in the test set (~13% of the total number of nanoparticles), the model for Split 2 contained 12 outliers in the training set and 5 in the test set (~19.5% of the total dataset), and the model for Split 3 contained 6 outliers in the training set and 1 in the test set (~8%). As data on zeta potentials is very sensitive, a variation in the number of outliers is related to a variation in measurement distributions in the collected database [36]. 

However, a high *RMSE* for validation sets (up to 17.2) reflects potential inaccuracies for the determination of stable/unstable nanoparticles. In other words, obtained models are useful for predictions of charge (positive/negative), but have only a limited usefulness for the purpose of stability prediction (nanoparticles with ξ < −20 and ξ > 20 are stable nanoparticles), due to the high deviation. More research is needed to further address these issues. It should be noted that the quality of any predictive model is the ability to adequately predict endpoints for external objects. In that case, the external prediction is invisible during model development [37]. At the same time, an excellent statistical quality of a model for the training set is often an indicator of overfitting [38]. In the present case (Table 4), the predictive potential (external prediction) is suitable, as overfitting is not observed.

**Figure 2:**
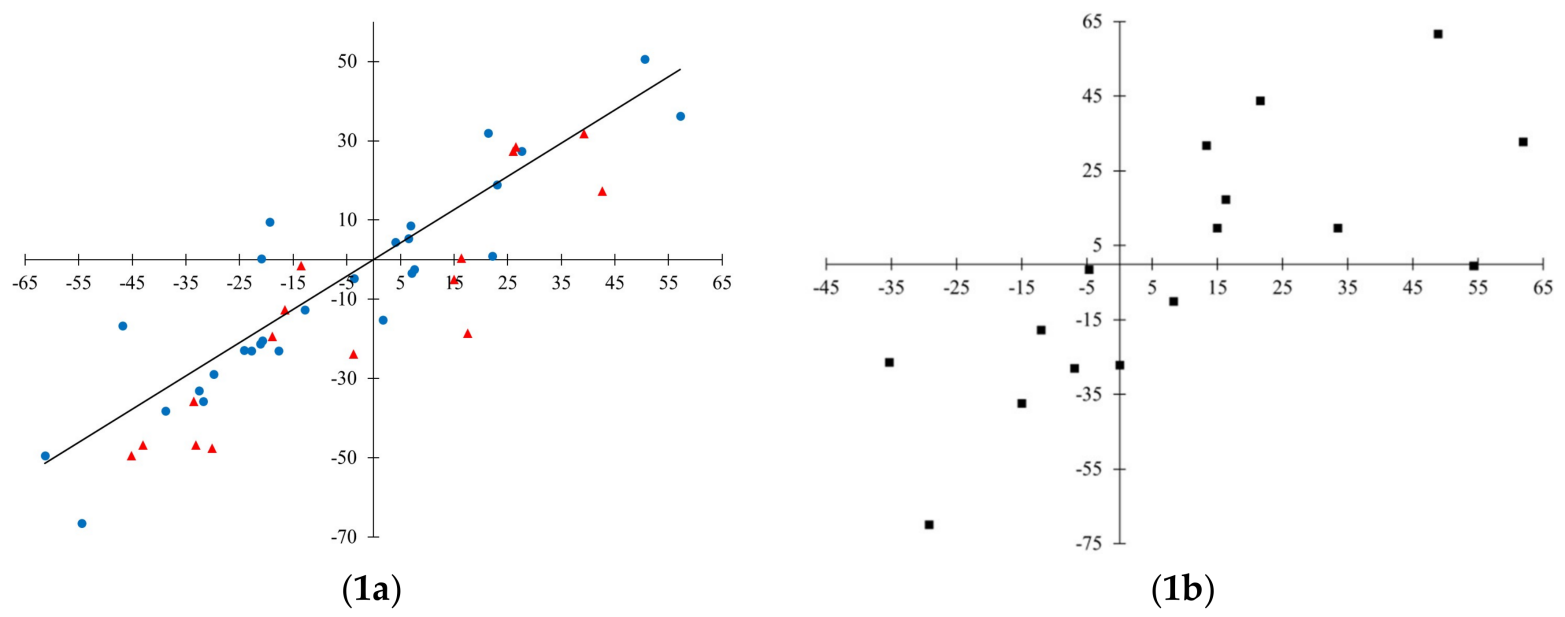

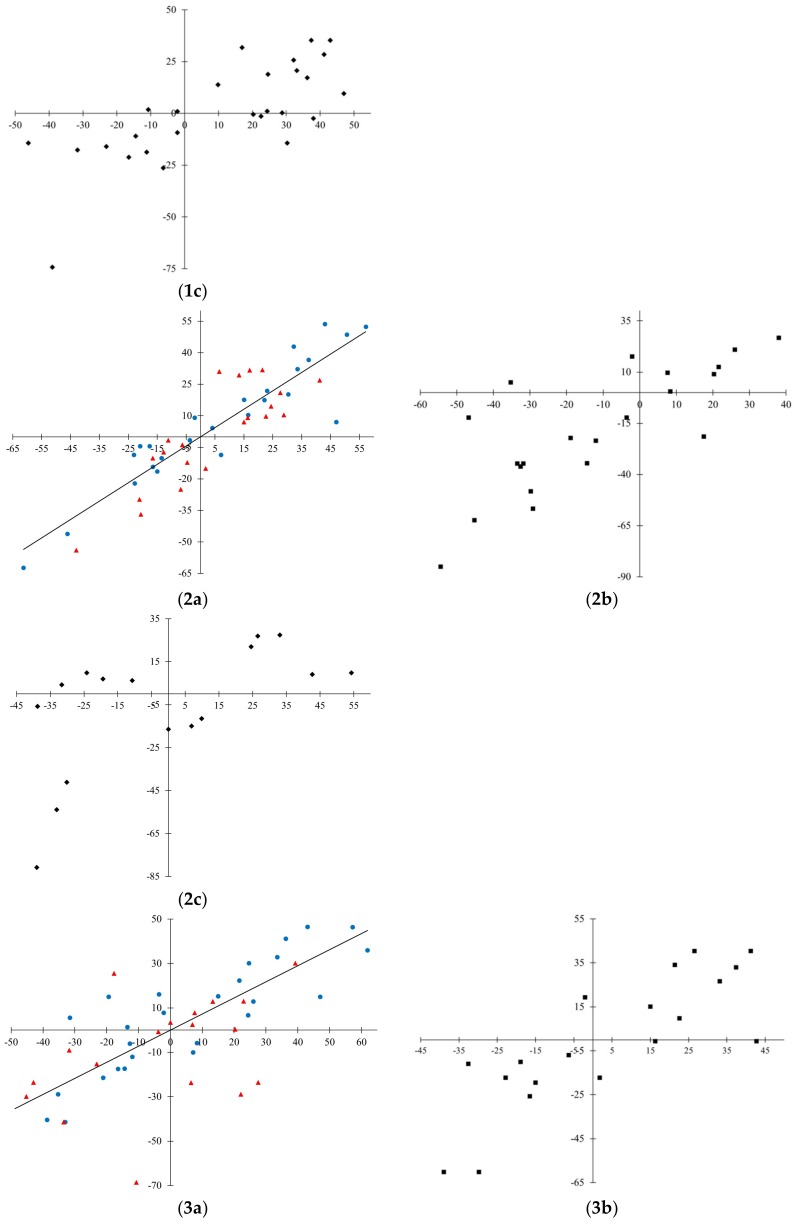

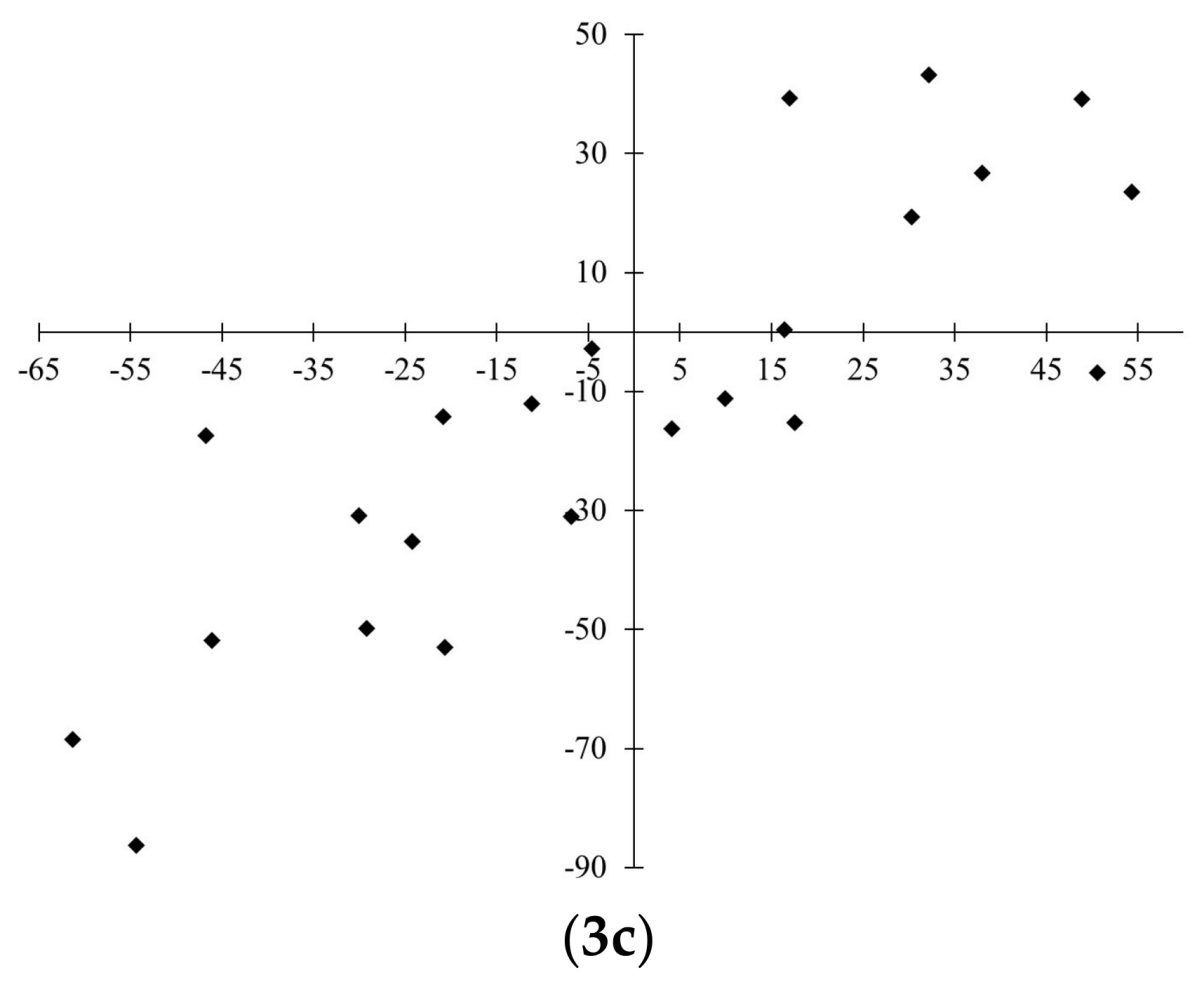
Observed-predicted diagrams for the developed models. Model 1: **1a**—training in blue, validation in red; **1b**—calibration; **1c**—invisible training. Model 2: **2a**—training in blue, validation in red; **2b**—calibration; **2c**—invisible training. Model 3: **3a**—training in blue, validation in red; **3b**—calibration; **3c**—invisible training.

Scatterplots for observed and predicted values are presented in Figure 2. In general, scattering of data for invisible training sets (Figure 2(1c, 2c, and 3c)) is quite significant. This can be explained by complexity for fitting data obtained from various sources. Among the three developed quasi-SMILES based models, the most reliable predictions for the invisible training set are considered to be those obtained for Model 3.

Each developed model could be represented in a linear form:

(6) ξ = 1.044(±0.524) + 13.666(±0.238)⋅DCW(1,30),

(7) ξ = −33.530(±0.596) + 11.319(±0.105)⋅DCW(1,7),

(8) ξ = −25.808(±0.649) + 16.732(±0.284)⋅DCW(1,23).

As mentioned, the DCW is calculated based on correlation weights for different attributes. An example of calculations for quasi-SMILES O=[Al]O[Al]=O%15%54 is presented in Table 4. The resultant DCW(1,30) value represents the summation of all correlation weights and is equal to 1.435.

Having data on several runs of the Monte Carlo optimization, one can select attributes of quasi-SMILES that have solely positive correlation weights. These attributes can be interpreted as promoters for the increase in zeta-potential. On the other hand, attributes of quasi-SMILES that have negative correlation weights in several runs of the optimization also could be extracted. Those can be interpreted as promoters for the decrease of zeta-potential. Table 5 contains examples of promoters for the increase or decrease zeta-potential.

## 4. Conclusions

The authors here have gathered experimental reports on zeta potential measurements of nano-sized metal oxides and analyzed collected data points, selecting for further studies only those that are reliable and comparable among different publications. In this study, a simple workflow was developed and applied, which allowed for the use of modeling methods even for quite complex data collected from different sources. Specific quasi-SMILES descriptors for the assessment of zeta potentials were calculated and tested. The presented quasi-SMILES descriptors directly take into account the size of nanoparticles, being capable of reflecting the size-dependent behavior of zeta potentials. At the same time, the developed descriptors do not require complex or long-term computations. The resulting models showed reasonable statistical characteristics. Thus, the general modeling workflow, due to its simplicity and transparency, can be applied for nano-QSAR modeling. The presented database can be used as a basis for extensive nano-QSPR modeling in the future. 

## Figures and Tables

**Table 1:** Experimental data on zeta potentials and ordinary SMILES codes.

#	NANOPARTICLE	SMILES	Nominal Size (nm)	Size in H_2_O (nm)	ζ in H_2_O
1	Al_2_O_3_	O=[Al]O[Al]=O	11.40	94.70	39.20
2	Al_2_O_3_	O=[Al]O[Al]=O	60.00	763.00	33.10
3	Al_2_O_3_	O=[Al]O[Al]=O	13.56	312.60	38.00
4	Al_2_O_3_	O=[Al]O[Al]=O	30.00	210.00	43.00
5	Al_2_O_3_	O=[Al]O[Al]=O	40.00	237.00	36.20
6	Al_2_O_3_	O=[Al]O[Al]=O	55.00	330.00	30.30
7	Bi_2_O_3_	O=[Bi]O[Bi]=O	144.00	4084.00	−16.50
8	CeO_2_	O=[Ce][Ce]=O	9.70	165.40	41.20
9	CeO_2_	O=[Ce][Ce]=O	13.04	200.70	26.50
10	CeO_2_	O=[Ce][Ce]=O	18.30	197.60	21.40
11	CeO_2_	O=[Ce][Ce]=O	8.00	2610.00	15.00
12	Co_3_O_4_	[Co]=O.O=[Co]O[Co]=O	11.50	99.20	23.00
13	Co_3_O_4_	[Co]=O.O=[Co]O[Co]=O	10.00	174.50	24.60
14	CoO	[Co]=O	71.80	184.80	21.60
15	CoO	[Co]=O	55.00	262.00	17.50
16	Cr_2_O_3_	O=[Cr]O[Cr]=O	193.00	256.40	−32.60
17	Cr_2_O_3_	O=[Cr]O[Cr]=O	47.00	426.00	−12.00
18	CuO	[Cu]=O	23.10	171.70	37.40
19	CuO	[Cu]=O	12.50	130.00	17.00
20	CuO	[Cu]=O	12.80	263.30	7.60
21	CuO	[Cu]=O	28.00	285.00	24.40
22	Dy_2_O_3_	O=[Dy]O[Dy]=O	6.00	565.20	50.60
23	Fe_2_O_3_	O=[Fe]O[Fe]=O	30.00	942.00	−22.80
24	Fe_2_O_3_	O=[Fe]O[Fe]=O	20.00	1565.00	−11.20
25	Fe_2_O_3_	O=[Fe]O[Fe]=O	12.30	144.70	−2.10
26	Fe_2_O_3_	O=[Fe]O[Fe]=O	68.00	6000.00	−6.30
27	Fe_3_O_4_	O=[Fe].O=[Fe]O[Fe]=O	9.50	128.00	22.10
28	Fe_3_O_4_	O=[Fe].O=[Fe]O[Fe]=O	20.00	685.00	−17.70
29	Fe_3_O_4_	O=[Fe].O=[Fe]O[Fe]=O	119.00	127.00	8.33
30	Fe_3_O_4_	O=[Fe].O=[Fe]O[Fe]=O	12.30	198.40	−2.10
31	Gd_2_O_3_	O=[Gd]O[Gd]=O	43.80	195.70	6.50
32	HfO_2_	O=[Hf]=O	28.40	291.80	33.50
33	In_2_O_3_	O=[In]O[In]=O	35.80	212.70	57.20
34	In_2_O_3_	O=[In]O[In]=O	59.60	192.20	61.90
35	In_2_O_3_	O=[In]O[In]=O	60.00	308.00	22.60
36	In_2_O_3_	O=[In]O[In]=O	17.00	391.00	−31.60
37	La_2_O_3_	O=[La]O[La]=O	24.60	211.00	54.30
38	La_2_O_3_	O=[La]O[La]=O	65.00	508.00	−3.60
39	MgO	O=[Mg]	13.60	1964.00	6.90
40	Mn_2_O_3_	O=[Mn]O[Mn]=O	51.50	268.80	−46.10
41	Mn_3_O_4_	O=[Mn]O[Mn]O[Mn]=O	15.20	395.00	−14.40
42	Ni_2_O_3_	O=[Ni]O[Ni]=O	140.60	311.40	32.20
43	NiO	[Ni]=O	5.30	209.70	48.90
44	NiO	[Ni]=O	30.00	1634.00	13.30
45	NiO	[Ni]=O	13.10	228.00	27.60
46	NiO	[Ni]=O	14.00	399.00	26.00
47	Sb_2_O_3_	O=[Sb]O[Sb]=O	20.80	125.00	−24.20
48	Sb_2_O_3_	O=[Sb]O[Sb]=O	11.80	147.60	−35.30
49	Sb_2_O_3_	O=[Sb]O[Sb]=O	84.00	619.00	−20.70
50	SiO_2_	O=[Si]=O	6.20	373.50	−29.20
51	SiO_2_	O=[Si]=O	7.80	148.00	−33.50
52	SiO_2_	O=[Si]=O	114.00	123.00	−43.00
53	SiO_2_	O=[Si]=O	13.50	113.40	−31.80
54	SiO_2_	O=[Si]=O	35.00	28.90	−23.10
55	SiO_2_	O=[Si]=O	51.00	52.90	−30.10
56	SiO_2_	O=[Si]=O	110.00	121.00	−33.10
57	SiO_2_	O=[Si]=O	420.00	703.00	−39.00
58	SiO_2_	O=[Si]=O	20.00	1230.00	−29.80
59	SnO_2_	O=[Sn]=O	62.40	203.70	−38.80
60	SnO_2_	O=[Sn]=O	15.00	3971.00	−21.10
61	TiO_2_	O=[Ti]=O	30.50	358.20	−16.50
62	TiO_2_	O=[Ti]=O	121.2	171.00	−13.50
63	TiO_2_	O=[Ti]=O	50.00	550.00	−18.90
64	TiO_2_	O=[Ti]=O	3.59	30.60	47.00
65	TiO_2_	O=[Ti]=O	108.00	117.00	−4.64
66	TiO_2_	O=[Ti]=O	12.60	166.00	−19.40
67	TiO_2_	O=[Ti]=O	10.00	116.00	15.00
68	TiO_2_	O=[Ti]=O	16.00	1500.00	7.09
69	TiO_2_	O=[Ti]=O	100.00	1510.00	4.07
70	TiO_2_	O=[Ti]=O	50.00	1610.00	1.77
71	TiO_2_	O=[Ti]=O	5.00	2710.00	−3.75
72	TiO_2_	O=[Ti]=O	42.00	748.00	−10.70
73	WO_3_	O=[W](=O)=O	10.60	62.80	−45.20
74	WO_3_	O=[W](=O)=O	16.60	176.60	−61.30
75	WO_3_	O=[W](=O)=O	15.90	545.50	−54.40
76	Y_2_O_3_	O=[Y]O[Y]=O	32.70	312.20	42.70
77	Y_2_O_3_	O=[Y]O[Y]=O	38.00	357.00	16.30
78	Yb_2_O_3_	O=[Yb]O[Yb]=O	61.70	230.70	9.90
79	ZnO	[Zn]=O	20.00	165.00	16.40
80	ZnO	[Zn]=O	30.00	501.00	−46.80
81	ZnO	[Zn]=O	20.00	759.00	0.017
82	ZnO	[Zn]=O	36.14	532.00	20.30
83	ZnO	[Zn]=O	22.60	204.50	28.80
84	ZnO	[Zn]=O	13.00	413.00	−15.00
85	ZnO	[Zn]=O	71.00	1614.00	−20.90
86	ZrO_2_	O=[Zr]=O	40.10	306.50	−12.80
87	ZrO_2_	O=[Zr]=O	27.00	2337.00	−6.90

**Table 2:** Correlation weights of attributes of quasi-SMILES for developed models.

SPLIT 1		SPLIT 2		SPLIT 3	
*A*_k_	CW(*A*_k_)	*A*_k_	CW(*A*_k_)	*A*_k_	CW(*A*_k_)
%11.........	0.80800	%11.........	1.69179	%11.........	0.37780
%12.........	1.06400	%12.........	2.12261	%12.........	0.00137
%13.........	2.25071	%13.........	4.00196	%13.........	1.24844
%14.........	−0.05794	%14.........	1.12049	%14.........	−0.05971
%15.........	1.99908	%15.........	2.18310	%15.........	0.62086
%16.........	2.49597	%17.........	2.81530	%16.........	0.00000
%17.........	1.37532	%18.........	0.00000	%17.........	0.00000
%18.........	0.00000	%19.........	0.18353	%18.........	−0.68769
%19.........	0.00000	%20.........	0.00261	%19.........	−0.43846
%20.........	0.00000	%21.........	1.55940	%20.........	0.00000
%24.........	−1.18511	%24.........	0.00000	%21.........	0.00000
%40.........	0.00000	%40.........	0.00000	%24.........	0.00000
%51.........	2.44195	%51.........	1.62637	%40.........	0.00000
%52.........	−0.06464	%52.........	−0.31720	%51.........	1.81351
%53.........	1.19226	%53.........	−0.37875	%52.........	0.24603
%54.........	0.43457	%54.........	−0.75096	%53.........	0.74900
%55.........	1.18553	%55.........	−1.56470	%54.........	0.00000
%57.........	2.68648	%57.........	0.00000	%55.........	0.00000
%58.........	1.49714	%58.........	0.25164	%57.........	0.00000
%59.........	0.00000	%60.........	0.00000	%58.........	0.31647
%60.........	−1.62667	%63.........	0.80912	%62.........	0.00000
%62.........	0.00000	%64.........	0.87088	%63.........	0.31311
%70.........	4.87656	%71.........	0.43610	%70.........	3.18594
%80.........	0.00000	(...........	−0.94127	%71.........	0.00000
(...........	−2.18987	=...........	−1.49711	%80.........	0.00000
=...........	0.37657	Al..........	1.93437	(...........	0.00000
Al..........	0.00000	Bi..........	−0.18907	=...........	−2.74671
Ce..........	−0.37460	Ce..........	1.06608	Al..........	1.25135
Co..........	−1.06410	Co..........	0.31118	Bi..........	0.00000
Cr..........	−0.12456	Cr..........	0.00000	Ce..........	1.12551
Cu..........	−0.75250	Cu..........	2.50088	Co..........	0.69227
Fe..........	−1.50240	Fe..........	0.18254	Cr..........	0.31466
Dy..........	1.31505	Dy..........	2.93269	Cu..........	1.94212
Gd..........	−1.68804	Hf..........	5.37088	Fe..........	0.24768
Hf..........	0.00000	O...........	0.19036	Dy..........	0.00000
O...........	−0.50138	In..........	0.93871	Hf..........	5.75412
In..........	−0.55827	La..........	0.00000	O...........	0.49842
La..........	−1.30825	Mg..........	0.00000	In..........	0.62411
Mg..........	1.62027	Mn..........	−0.99685	La..........	0.56389
Mn..........	0.00000	Ni..........	3.49511	Mn..........	−0.87592
Ni..........	1.43678	W...........	−1.31268	Ni..........	1.93411
W...........	−2.06470	Sb..........	0.00000	W...........	0.00000
Sb..........	−2.12939	Si..........	−2.06057	Sb..........	−1.19208
Si..........	−5.56503	Y...........	0.00000	Si..........	0.43778
Y...........	0.00000	Sn..........	0.00000	Y...........	0.00000
Sn..........	−6.93877	Ti..........	1.62433	Sn..........	−0.81116
Ti..........	−2.25298	[...........	0.62653	Ti..........	2.74678
[...........	−0.06193	^...........	0.62086	[...........	1.00212
^...........	2.49929	Yb..........	0.00000	^...........	−0.69017
Yb..........	0.00000	Zn..........	0.19212	Yb..........	0.00000
Zn..........	−3.30812	-	-	Zn..........	0.00000
Zr..........	−2.81327	-	-	Zr..........	2.18349

**Table 3:** The statistical characteristics of developed models.

SPLIT	Set	*n*	*R*^2^	*CCC*	*q*^2^	*RMSE*	*MAE*	*F*
1	training	28	0.8257	0.9045	0.7993	12.2	7.71	123
	invisible training	27	0.5511	0.6809	0.4812	21.2	16.7	31
	calibration	16	0.5888	0.7065	0.4950	24.8	19.7	20
	validation	16	0.8213	-	0.7814	15.8	11.6	-
2	training	25	0.8668	0.9286	0.8518	11.3	7.06	150
	invisible training	20	0.5258	0.7195	0.4508	25.6	21.4	20
	calibration	21	0.6121	0.7583	0.5461	20.2	15.5	30
	validation	21	0.7268	-	0.6694	13.1	11.7	-
3	training	26	0.7139	0.8331	0.6802	15.9	11.2	60
	invisible training	23	0.7139	0.7968	0.6679	22.9	18.1	52
	calibration	19	0.6789	0.8078	0.6259	17.7	13.4	36
	validation	19	0.6707	-	0.6112	17.2	14.7	-

**Table 4:** Attribute calculation of DCW(1,30) for O=[Al]O[Al]=O%15%54.

Attributes of Quasi-SMILES, *A*_k_	CW(*A*)	*N*_t_	*N*_i_	*N*_c_
O...........	−0.5014	28	27	16
=...........	0.3766	28	27	16
[...........	−0.0619	28	27	16
Al..........	0.000	0	5	0
[...........	−0.0619	28	27	16
O...........	−0.5014	28	27	16
[...........	−0.0619	28	27	16
Al..........	0.000	0	5	0
[...........	−0.0619	28	27	16
=...........	0.3766	28	27	16
O...........	−0.5014	28	27	16
%15.........	1.9991	3	4	2
%54.........	0.4346	1	3	1

CW(*A*) is the correlation weight for certain quasi-SMILES attribute, *N*_t_ is the number of *A* in the training set, *N*_i_ is the number of *A* in the invisible training set, and *N*_c_ is the number of *A* in the calibration set. DCW: descriptor of correlation weights.

**Table 5:** Factors related to the increase (positives) of to the decrease (negatives) of zeta-potentials.

ATTRIBUTES OF QUASI-SMILES, *A*_K_	CW(*A*_k_) RUN 1	CW(*A_k_*) RUN 2	CW(*A*_k_) RUN 3
SPLIT 1			
%11.........	0.56499	0.30946	0.68722
%51.........	3.24897	2.25246	2.62163
%12.........	0.99548	0.56358	0.99608
%53.........	1.62537	0.94188	1.43373
%58.........	1.81634	1.25070	1.56032
%13.........	2.49862	1.69067	2.18703
%15.........	2.37600	1.43252	2.00299
%16.........	2.93845	2.25077	2.55826
%17.........	1.31143	0.87860	1.37092
O...........	−1.37959	−0.37307	−0.25319
%52.........	−0.24995	−0.18750	−0.12144
Ti..........	−2.12736	−2.30889	−2.18847
Fe..........	−2.12005	−1.50302	−1.62577
(...........	−1.99668	−2.18891	−1.37159
W...........	−2.62409	−2.12897	−3.37530
Sb..........	−2.93941	−2.31014	−2.43815
Si..........	−6.87065	−5.75002	−5.87985
Sn..........	−8.68861	−7.12619	−7.37931
Zn..........	−4.87376	−3.50015	−4.37858
SPLIT 2			
[...........	1.80915	2.24881	0.87914
%51.........	2.49518	2.81286	1.87221
%11.........	1.56337	2.24885	1.68514
%12.........	1.99713	2.68668	2.56602
%52.........	0.05800	0.12815	0.12410
Ti..........	2.12419	3.31268	1.93265
%58.........	0.18888	0.12581	0.12043
%13.........	4.12763	4.74576	4.18721
%14.........	1.18675	0.87720	1.00481
Al..........	1.87368	2.37543	2.18986
=...........	−2.25434	−1.87704	−2.00105
%19.........	−0.62262	−0.99988	−0.43452
%54.........	−0.24929	−0.18668	−0.74893
%55.........	−1.30860	−1.00469	−2.49819
(...........	−1.12284	−1.43542	−1.18555
Mn..........	−1.74769	−2.25123	−1.74980
W...........	−0.37024	−0.74538	−0.87798
Si..........	−1.25313	−1.50202	−1.87854
SPLIT 3			
[...........	1.12589	0.74879	1.75183
%51.........	1.62591	1.37040	1.99838
%11.........	0.62966	0.37865	0.81600
%52.........	0.19018	0.12505	0.06011
Ti..........	2.81440	2.30878	3.56507
%12.........	0.37221	0.12249	0.44184
%15.........	1.06671	0.62861	1.18785
%13.........	1.68571	1.12890	1.99793
In..........	0.99791	0.74823	0.94120
Al..........	1.55896	1.24856	1.74813
=...........	−1.68634	−1.93897	−2.18763
%19.........	−0.24851	−0.37406	−0.12216
Sn..........	−1.06603	−0.74729	−0.87290
%18.........	−0.68596	−0.74839	−0.68558
Mn..........	−0.55928	−0.49779	−1.00283
Sb..........	−0.87118	−0.74661	−1.30958

## Supplementary Materials

The following are available online at http://www.mdpi.com/2079-4991/8/4/243/s1.
