# Supplementary material for: Towards the Development of Global Nano-Quantitative Structure–Property Relationship Models: Zeta Potentials of Metal Oxide Nanoparticles
Source: Nanomaterials (Basel). 2018 Apr 15;8(4):243. doi: 10.3390/nano8040243 (PMC5923573; doi:10.3390/nano8040243)
Supplement: Supplementary file 1 [file nanomaterials-08-00243-s001.pdf]

Toward the Development of Global Nano-Quantitative Structure–Property Relationship Models: Zeta Potentials of Metal Oxide Nanoparticles

Andrey A. Toropov [1], Natalia Sizochenko [2,3], Alla P. Toropova [1] and Jerzy Leszczynski [2,*]

Table S1. Definition of attribute of quasi-SMILES for nominal sizes.

Range (in nm)		Number of samples in range	Quasi-SMILES element
From	To		
3.590	17.470	33	%11
17.470	31.351	18	%12
31.351	45.231	9	%13
45.231	59.111	7	%14
59.111	72.992	9	%15
72.992	86.872	1	%16
86.872	100.752	1	%17
-	114.633	3	%18
114.633	128.513	2	%19
128.513	142.393	1	%20
142.393	156.274	1	%21
156.274	170.154	0	%22
170.154	184.034	0	%23
184.034	197.915	1	%24
197.915	211.795	0	%25
211.795	225.675	0	%26
225.675	239.556	0	%27
239.556	253.436	0	%28
253.436	267.316	0	%29
267.316	281.197	0	%30
-	295.077	0	%31
295.077	308.957	0	%32
308.957	322.838	0	%33
322.838	336.718	0	%34
336.718	350.598	0	%35
350.598	364.479	0	%36
364.479	378.359	0	%37
378.359	392.239	0	%38
392.239	406.120	0	%39
406.120	420.000	1	%40

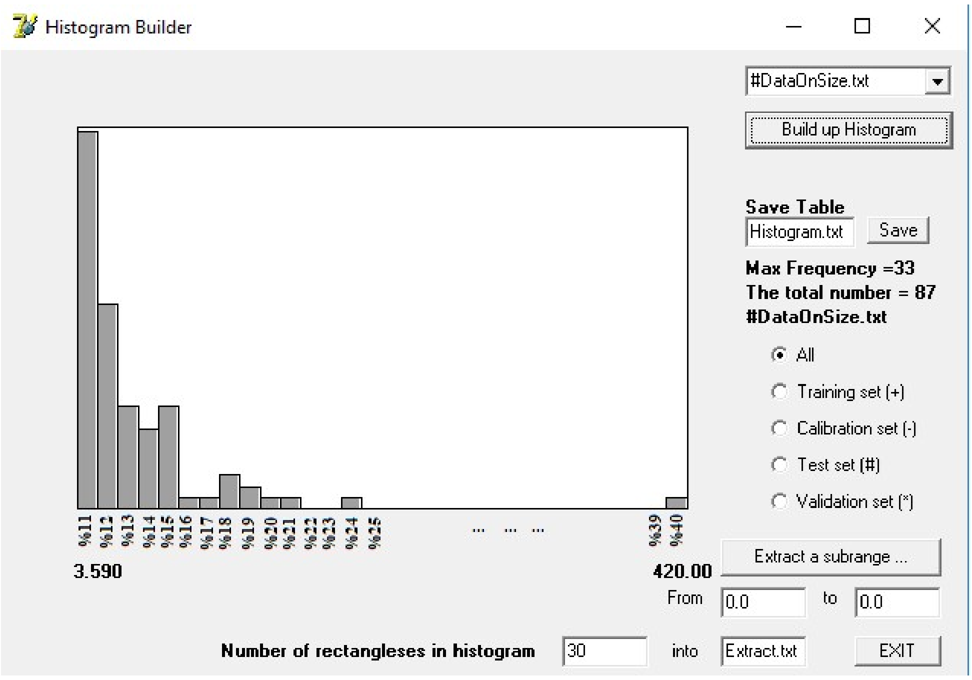

Figure S1. Distribution of quasi-SMILES attributes for nominal sizes.

Table S2. Definition of attribute of quasi-SMILES for sizes in H_2O.

Range (in nm)		Number of samples in range	Quasi-SMILES element
From	From		
28.900	227.937	37	%51
227.937	426.973	23	%52
426.973	626.010	7	%53
626.010	825.047	5	%54
825.047	1024.083	1	%55
1024.083	1223.120	0	%56
1223.120	1422.157	1	%57
1422.157	1621.193	5	%58
1621.193	1820.230	1	%59
1820.230	2019.267	1	%60
2019.267	2218.303	0	%61
2218.303	2417.340	1	%62
2417.340	2616.377	1	%63
2616.377	2815.413	1	%64
2815.413	3014.450	0	%65
3014.450	3213.487	0	%66
3213.487	3412.523	0	%67
3412.523	3611.560	0	%68
3611.560	3810.597	0	%69
3810.597	4009.633	1	%70
4009.633	4208.670	1	%71
4208.670	4407.707	0	%72
4407.707	4606.743	0	%73
4606.743	4805.780	0	%74
4805.780	5004.817	0	%75
5004.817	5203.853	0	%76
5203.853	5402.890	0	%77
5402.890	5601.927	0	%78

5601.927	5800.963	0	%79
5800.963	6000.000	1	%80

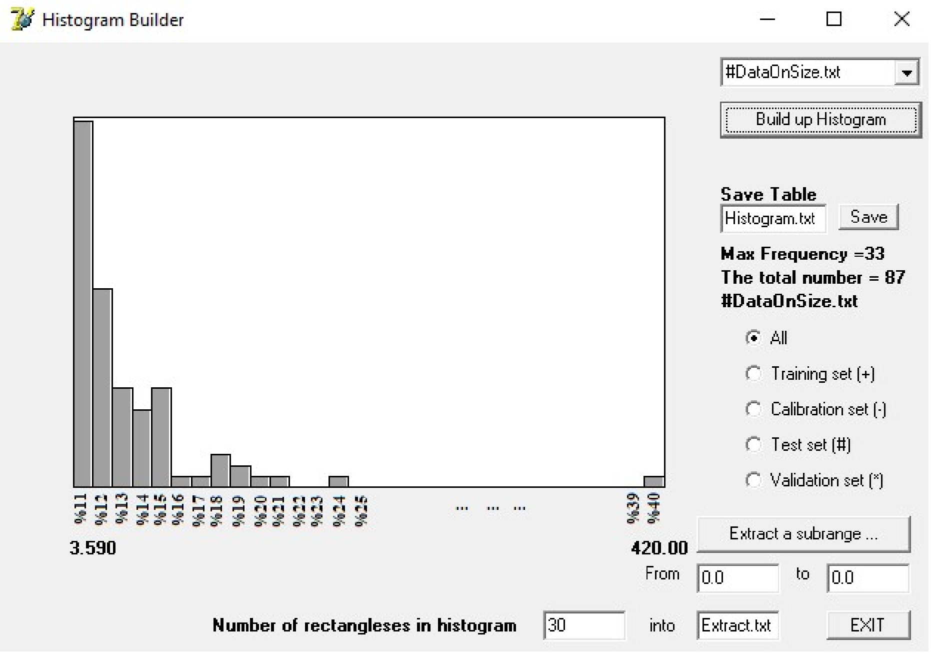

Figure S2. Distribution of quasi-SMILES attributes for sizes in media.

Table S3. Three splits into the training (+), invisible training (-), calibration (#), and validation sets.

SPLIT 1	SPLIT 2	SPLIT 3
*1. O=[Al]O[Al]=O%11%51	-1. O=[Al]O[Al]=O%11%51	*1. O=[Al]O[Al]=O%11%51
-2. O=[Al]O[Al]=O%15%54	-2. O=[Al]O[Al]=O%15%54	#2. O=[Al]O[Al]=O%15%54
-3. O=[Al]O[Al]=O%11%52	#3. O=[Al]O[Al]=O%11%52	-3. O=[Al]O[Al]=O%11%52
-4. O=[Al]O[Al]=O%12%51	+4. O=[Al]O[Al]=O%12%51	+4. O=[Al]O[Al]=O%12%51
-5. O=[Al]O[Al]=O%13%52	-5. O=[Al]O[Al]=O%13%52	+5. O=[Al]O[Al]=O%13%52
-6. O=[Al]O[Al]=O%14%52	+6. O=[Al]O[Al]=O%14%52	-6. O=[Al]O[Al]=O%14%52
*7. O=[Bi]O[Bi]=O%21%71	+7. O=[Bi]O[Bi]=O%21%71	#7. O=[Bi]O[Bi]=O%21%71
-8. O=[Ce][Ce]=O%11%51	*8. O=[Ce][Ce]=O%11%51	#8. O=[Ce][Ce]=O%11%51
*9. O=[Ce][Ce]=O%11%51	-9. O=[Ce][Ce]=O%11%51	#9. O=[Ce][Ce]=O%11%51
+10. O=[Ce][Ce]=O%12%	*10. O=[Ce][Ce]=O%12%51	#10. O=[Ce][Ce]=O%12%51
*11. O=[Ce][Ce]=O%11%63	+11. O=[Ce][Ce]=O%11%63	+11. O=[Ce][Ce]=O%11%63
+12. [Co]=O.O=[Co]O[Co]=O%11%51	+12. [Co]=O.O=[Co]O[Co]=O%11%51	*12. [Co]=O.O=[Co]O[Co]=O%11%51
-13. [Co]=O.O=[Co]O[Co]=O%11%51	-13. [Co]=O.O=[Co]O[Co]=O%11%51	+13. [Co]=O.O=[Co]O[Co]=O%11%51
#14. [Co]=O%15%51	#14. [Co]=O%15%51	+14. [Co]=O%15%51
*15. [Co]=O%14%52	#15. [Co]=O%14%52	-15. [Co]=O%14%52
+16. O=[Cr]O[Cr]=O%24%52	#16. O=[Cr]O[Cr]=O%24%52	#16. O=[Cr]O[Cr]=O%24%52
#17. O=[Cr]O[Cr]=O%14%52	#17. O=[Cr]O[Cr]=O%14%52	+17. O=[Cr]O[Cr]=O%14%52
-18. [Cu]=O%12%51	+18. [Cu]=O%12%51	#18. [Cu]=O%12%51
-19. [Cu]=O%11%51	*19. [Cu]=O%11%51	-19. [Cu]=O%11%51
+20. [Cu]=O%11%52	#20. [Cu]=O%11%52	*20. [Cu]=O%11%52
-21. [Cu]=O%12%52	*21. [Cu]=O%12%52	+21. [Cu]=O%12%52
+22. O=[Dy]O[Dy]=O%11%53	+22. O=[Dy]O[Dy]=O%11%53	-22. O=[Dy]O[Dy]=O%11%53
+23. O=[Fe]O[Fe]=O%12%55	+23. O=[Fe]O[Fe]=O%12%55	#23. O=[Fe]O[Fe]=O%12%55
-24. O=[Fe]O[Fe]=O%12%58	*24. O=[Fe]O[Fe]=O%12%58	-24. O=[Fe]O[Fe]=O%12%58
-25. O=[Fe]O[Fe]=O%11%51	+25. O=[Fe]O[Fe]=O%11%51	#25. O=[Fe]O[Fe]=O%11%51
-26. O=[Fe]O[Fe]=O%15%80	*26. O=[Fe]O[Fe]=O%15%80	#26. O=[Fe]O[Fe]=O%15%80
+27. O=[Fe].O=[Fe]O[Fe]=O%11%51	+27. O=[Fe].O=[Fe]O[Fe]=O%11%51	*27. O=[Fe].O=[Fe]O[Fe]=O%11%51
+28. O=[Fe].O=[Fe]O[Fe]=O%12%54	+28. O=[Fe].O=[Fe]O[Fe]=O%12%54	*28. O=[Fe].O=[Fe]O[Fe]=O%12%54
#29. O=[Fe].O=[Fe]O[Fe]=O%19%51	#29. O=[Fe].O=[Fe]O[Fe]=O%19%51	+29. O=[Fe].O=[Fe]O[Fe]=O%19%51
-30. O=[Fe].O=[Fe]O[Fe]=O%11%51	#30. O=[Fe].O=[Fe]O[Fe]=O%11%51	+30. O=[Fe].O=[Fe]O[Fe]=O%11%51

+31. O=[Gd]O[Gd]=O%13%51	*31. O=[Gd]O[Gd]=O%13%51 6.500	*31. O=[Gd]O[Gd]=O%13%51
#32. O=[Hf]=O%12%52	+32. O=[Hf]=O%12%52	+32. O=[Hf]=O%12%52
+33. O=[In]O[In]=O%13%51	+33. O=[In]O[In]=O%13%51	+33. O=[In]O[In]=O%13%51
#34. O=[In]O[In]=O%15%51	-34. O=[In]O[In]=O%15%51	+34. O=[In]O[In]=O%15%51
-35. O=[In]O[In]=O%15%52	*35. O=[In]O[In]=O%15%52	#35. O=[In]O[In]=O%15%52
-36. O=[In]O[In]=O%11%52	-36. O=[In]O[In]=O%11%52	+36. O=[In]O[In]=O%11%52
#37. O=[La]O[La]=O%12%51	-37. O=[La]O[La]=O%12%51	-37. O=[La]O[La]=O%12%51
+38. O=[La]O[La]=O%15%53	#38. O=[La]O[La]=O%15%53	+38. O=[La]O[La]=O%15%53
+39. O=[Mg]%11%60	-39. O=[Mg]%11%60	*39. O=[Mg]%11%60
-40. O=[Mn]O[Mn]=O%14%52	+40. O=[Mn]O[Mn]=O%14%52	-40. O=[Mn]O[Mn]=O%14%52
-41. O=[Mn]O[Mn]O[Mn]=O%11%52	#41. O=[Mn]O[Mn]O[Mn]=O%11%52	+41. O=[Mn]O[Mn]O[Mn]=O%11%52
-42. O=[Ni]O[Ni]=O%20%52	+42. O=[Ni]O[Ni]=O%20%52	-42. O=[Ni]O[Ni]=O%20%52
#43. [Ni]=O%11%51	-43. [Ni]=O%11%51	-43. [Ni]=O%11%51
#44. [Ni]=O%12%59	*44. [Ni]=O%12%59	*44. [Ni]=O%12%59
+45. [Ni]=O%11%52	*45. [Ni]=O%11%52	*45. [Ni]=O%11%52
*46. [Ni]=O%11%52	#46. [Ni]=O%11%52	+46. [Ni]=O%11%52
+47. O=[Sb]O[Sb]=O%12%51	-47. O=[Sb]O[Sb]=O%12%51	-47. O=[Sb]O[Sb]=O%12%51
#48. O=[Sb]O[Sb]=O%11%51	#48. O=[Sb]O[Sb]=O%11%51	+48. O=[Sb]O[Sb]=O%11%51
+49. O=[Sb]O[Sb]=O%16%53	*49. O=[Sb]O[Sb]=O%16%53	-49. O=[Sb]O[Sb]=O%16%53
#50. O=[Si]=O%11%52	#50. O=[Si]=O%11%52	-50. O=[Si]=O%11%52
*51. O=[Si]=O%11%51	#51. O=[Si]=O%11%51	*51. O=[Si]=O%11%51
*52. O=[Si]=O%18%51	*52. O=[Si]=O%18%51	*52. O=[Si]=O%18%51
+53. O=[Si]=O%11%51	#53. O=[Si]=O%11%51	*53. O=[Si]=O%11%51
-54. O=[Si]=O%13%51	+54. O=[Si]=O%13%51	*54. O=[Si]=O%13%51
*55. O=[Si]=O%14%51	-55. O=[Si]=O%14%51	-55. O=[Si]=O%14%51
*56. O=[Si]=O%18%51	-56. O=[Si]=O%18%51	+56. O=[Si]=O%18%51
-57. O=[Si]=O%40%54	-57. O=[Si]=O%40%54	#57. O=[Si]=O%40%54
+58. O=[Si]=O%12%57	#58. O=[Si]=O%12%57	#58. O=[Si]=O%12%57
+59. O=[Sn]=O%15%51	-59. O=[Sn]=O%15%51	+59. O=[Sn]=O%15%51
+60. O=[Sn]=O%11%70	*60. O=[Sn]=O%11%70	+60. O=[Sn]=O%11%70
-61. O=[Ti]=O%12%52	*61. O=[Ti]=O%12%52	+61. O=[Ti]=O%12%52
*62. O=[Ti]=O%19%51	+62. O=[Ti]=O%19%51	+62. O=[Ti]=O%19%51
*63. O=[Ti]=O%14%53	#63. O=[Ti]=O%14%53	#63. O=[Ti]=O%14%53
-64. O=[Ti]=O%11%51	+64. O=[Ti]=O%11%51	+64. O=[Ti]=O%11%51
#65. O=[Ti]=O%18%51	*65. O=[Ti]=O%18%51	-65. O=[Ti]=O%18%51
+66. O=[Ti]=O%11%51	-66. O=[Ti]=O%11%51	+66. O=[Ti]=O%11%51
#67. O=[Ti]=O%11%51	*67. O=[Ti]=O%11%51	#67. O=[Ti]=O%11%51
+68. O=[Ti]=O%11%58	+68. O=[Ti]=O%11%58	+68. O=[Ti]=O%11%58
+69. O=[Ti]=O%17%58	+69. O=[Ti]=O%17%58	-69. O=[Ti]=O%17%58
+70. O=[Ti]=O%14%58	*70. O=[Ti]=O%14%58	#70. O=[Ti]=O%14%58
*71. O=[Ti]=O%11%64	+71. O=[Ti]=O%11%64	*71. O=[Ti]=O%11%64
-72. O=[Ti]=O%13%54	-72. O=[Ti]=O%13%54	*72. O=[Ti]=O%13%54
*73. O=[W](=O)=O%11%51	#73. O=[W](=O)=O%11%51	*73. O=[W](=O)=O%11%51
+74. O=[W](=O)=O%11%51	+74. O=[W](=O)=O%11%51	-74. O=[W](=O)=O%11%51
+75. O=[W](=O)=O%11%53	#75. O=[W](=O)=O%11%53	-75. O=[W](=O)=O%11%53
*76. O=[Y]O[Y]=O%13%52	-76. O=[Y]O[Y]=O%13%52	#76. O=[Y]O[Y]=O%13%52
#77. O=[Y]O[Y]=O%13%52	*77. O=[Y]O[Y]=O%13%52	#77. O=[Y]O[Y]=O%13%52
-78. O=[Yb]O[Yb]=O%15%52	-78. O=[Yb]O[Yb]=O%15%52	-78. O=[Yb]O[Yb]=O%15%52
*79. [Zn]=O%12%51	+79. [Zn]=O%12%51	-79. [Zn]=O%12%51
+80. [Zn]=O%12%53	#80. [Zn]=O%12%53	-80. [Zn]=O%12%53
#81. [Zn]=O%12%54	-81. [Zn]=O%12%54	*81. [Zn]=O%12%54
-82. [Zn]=O%13%53	#82. [Zn]=O%13%53	*82. [Zn]=O%13%53
-83. [Zn]=O%12%51	*83. [Zn]=O%12%51	*83. [Zn]=O%12%51
#84. [Zn]=O%11%52	+84. [Zn]=O%11%52	#84. [Zn]=O%11%52
+85. [Zn]=O%15%58	+85. [Zn]=O%15%58	-85. [Zn]=O%15%58
+86. O=[Zr]=O%13%52	*86. O=[Zr]=O%13%52	+86. O=[Zr]=O%13%52
#87. O=[Zr]=O%12%62	*87. O=[Zr]=O%12%62	-87. O=[Zr]=O%12%62
